# Effect of Multi-Path Asynchronous Rolling Process on Microstructure and Mechanical Properties of ZK60 Magnesium Alloy

**DOI:** 10.3390/ma17071647

**Published:** 2024-04-03

**Authors:** Peng Jiang, Dawen Liu, Haixin Zou, Jianfu Liu, Wangping Wu, Haijun Pan, Zhizhi Wang, Yi Zhang, Guohong Dai

**Affiliations:** 1 School of Mechanical Engineering and Rail Transit, Changzhou University, Changzhou 213164, China; 2200360210@smail.cczu.edu.cn (H.Z.); 2200360120@smail.cczu.edu.cn (J.L.); wuwping@cczu.edu.cn (W.W.); phj@cczu.edu.cn (H.P.); zzwang@cczu.edu.cn (Z.W.); zy@cczu.edu.cn (Y.Z.); 2 School of Mechanical Engineering, Changzhou University, Changzhou 213164, China; s21050802010@smail.cczu.edu.cn

**Keywords:** ZK60 magnesium alloy, multi-path asynchronous rolling, texture and microstructure, mechanical properties

## Abstract

At the initial rolling temperature of 400 °C, ZK60 magnesium alloy was hot rolled by three different rolling paths with different roll speed ratios (RSR) of 1:1.15, 1:1.2, and 1:1.5, respectively. The effects of different rolling processes on the microstructure and mechanical properties of the alloy were studied. The microstructure, plasticity, strength, hardness, and texture intensity of rolled samples were analyzed in this work. The results show that the microstructure uniformity of the alloy under multi-path asynchronous rolling (MAR) is significantly improved, which improves the mechanical properties of the material to a certain extent, and effectively weakens the texture intensity of the basal plane and reduces the anisotropy. The amount of randomly oriented grains of ZK60 magnesium alloy rolled by the C-1.5 (path C combined with the RSR of 1:1.5) process are significantly increased, which significantly weakens the basal texture and improves the ductility of the alloy, greatly enhancing the processing and formability of ZK60 magnesium alloy.

## 1. Introduction

As the lightest magnesium metal material in current engineering applications, it has the advantages of low density, high specific strength, high specific stiffness, excellent shock absorption performance, and easy machining. With the rapid development of the automotive industry and modern rail transit, higher requirements are put forward for reducing product weight to achieve energy conservation and emission reduction. The application of magnesium alloy meets the requirements for the lightweight development of automobile and rail transit equipment [1,2,3]. However, due to its hexagonal close-packed (hcp) crystal structure, magnesium alloy only has a basal slip system at room temperature, which leads to difficulty of deformation at room temperature and cold forming [4,5,6].

At present, a large number of studies and reports have been conducted on the improvement of processing formability and mechanical properties of magnesium alloys, mainly through grain refinement and new preparation technologies. Rolling is an important processing method of magnesium alloy sheet. Xin et al. [7] studied the effects of the asynchronous rolling process on the microstructure, mechanical properties, and texture intensity of AZ31B alloys. The results showed that the AZ31B alloys sheet after asynchronous rolling had lower yield strength and higher elongation. Wang et al. [8] studied the evolution of microstructure and texture of ZK60 alloy during single-pass rolling, and the results showed that continuous dynamic recrystallization significantly refined the grain and improved the uniformity of the microstructure. However, Li et al. [9] found that the rolling path had no significant effect on the grain size change of alloys when studying the effect of the rolling path on the microstructure and properties of the ZK60 alloys sheet. After large plastic deformation, the grains of AZ31B alloy undergo preferential orientation, anisotropy is enhanced, and strong basal texture intensity is formed. At this time, the grains are in a hard orientation, and basal slip is difficult to carry out [10,11,12,13,14,15].

The question is how to reduce the texture intensity while ensuring the grain size get refined. Chen et al. [16] developed the asynchronous cross-rolling process to carry out experiments on AZ31 alloys. The results show that different roll speed ratios (RSR) can obtain different degrees of grain refinement effect, and the rolling path also has a certain impact on the microstructure and properties. Lin et al. [17] combined 90° cross-rolling and asynchronous rolling to deform tantalum. They found that due to the existence of “cross shear zone”, the equivalent strain and shear deformation acting on the material were increased, the uniformity of the microstructure and orientation distribution of deformed grains on the thickness was improved, the grain orientation distribution was relatively random, and the texture intensity of the tantalum sheet was significantly weakened. The rolling temperature also has a certain effect on the plastic deformation of the alloy. The recrystallization temperature of ZK60 magnesium alloy is 250 °C. Relevant studies [18,19,20] show that at higher temperatures, more non-base slip systems will be activated, which improves the machinability of magnesium alloy. Samman et al. [21], studying the effect of temperature on dynamic recrystallization of AZ31 magnesium alloy during deformation, found that compared with the lower deformation temperature, AZ31 magnesium alloy can produce the required complete recrystallization microstructure (small average grain size and almost random crystal structure) even after a small strain rate at 400 °C. In this paper, the effects of different rolling paths and different RSR on the microstructure and mechanical properties of ZK60 magnesium alloy were studied by the MAR process at 400 °C.

## 2. Materials and Methods

### 2.1. Processing Routes of Multi-Path Asynchronous Rolling (MAR)

At the initial rolling temperature of 400 °C, the experiments were completed through three different rolling paths with three RSR of 1:1.15, 1:1.2, and 1:1.5. The rolling paths are shown in Table 1.

Path A: During nine passes rolling, the rolling direction does not change during the experiment.

Path B: Turn 90° in the normal direction of the sheet before each next pass of rolling, and turn the plate 180° at the same time.

Path C: Turn 45° in the normal direction of the sheet before each next pass of rolling, and turn the plate 180° at the same time.

Before the next pass of rolling, the ZK60 alloy sample is quickly put into the muffle furnace and heated for 10 min to ensure the same rolling temperature 400 °C. Each sample is subject to a total of 9 passes of rolling test, and the preset reduction of each pass is 20%. In this test, MAR was carried out by ZK-W58A-3 asynchronous rolling mill (ZHIKE, Dongguan, China). The upper and lower roll diameters of the rolling mill were the same, and the upper and lower rolls rotated independently through two adjustable speed AC motors. During the experiment, MAR was realized by adjusting the rotation speed of the upper and lower rolls and changing the direction and contact surface of the plate entering the roll. The diameter of the upper and lower rollers was 200 mm; the total power was 45 KW; the speed of the upper and lower rollers could be adjusted within the range of 0–17 rpm. In this experiment, the upper roller was set as a constant speed, and the linear speed was 12 rpm. By adjusting the rotation speed of the lower roller, the required RSR could be obtained.

### 2.2. Materials Fabrication and Characterization

In this experiment, ZK60 alloy sheets with sizes of 200 mm × 150 mm × 3 mm (marked as received state) prepared by rolling method were selected as the research objects, and the alloy composition is shown in Table 2. ZK60 alloy was annealed in muffle furnace at 400 °C for 10 h, followed by air cooling [6]. After MAR deformation, specimens with dimensions 5 × 5 × t (mm^3^) were obtained by wire cutting, where t equals to the actual thickness (mm) depending on the rolling parameters, and the specimens were inlaid, burnished, polished, and finally chemically etched with a solution of concentrated nitric acid (1 g), glacial acetic acid (1 g), oxalic acid (1 g), and deionized water (100 mL). VHF-700F (Wanke Science Instrument, Xuzhou, China) type super depth-of-field microscope (OM), ZEISS Gemini300 scanning electron microscope (SEM, Jena, Germany), and OXFORD symmetry electron backscatter diffraction (EBSD, Abingdon upon Thames, UK) were used to study the microstructure characteristics of the samples, including microstructure uniformity, grain size and morphology, and texture intensity. The grain size was calculated using Nano Measure 1.2 software.

### 2.3. Testing of Mechanical Properties of Materials

**Figure 1:**
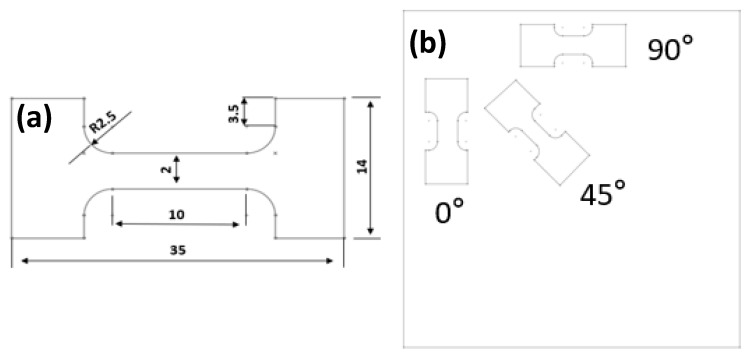
(**a**) Tensile sample size (unit: mm). (**b**) Sampling method of MARed ZK60 alloys.

After rolling, wire cutting was used to prepare tensile samples, with the dimensions shown in Figure 1a. TCL-T220 (SUNS, Shenzhen, China) microcomputer-controlled electronic universal testing machine was used to carry out tensile test of the alloy at room temperature. The sampling method is shown in Figure 1b, taking the 0° direction (taking the rolling direction of path a as the reference standard), 45° direction, and 90° direction of the rolled sheet, respectively. The hardness of the alloys was tested by HVS-1000 hardness tester (Laryee Technology Co., Ltd., Beijing, China).

## 3. Results

### 3.1. Microstructures of MARed ZK60 Magnesium Alloys

**Figure 2:**
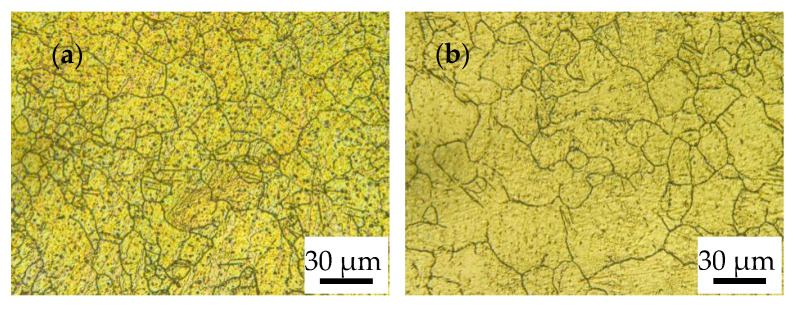
OM of ZK60 alloy: (**a**) received; (**b**) annealed.

Figure 2 shows the optical microstructures (OM) of received and annealed ZK60 alloys. It can be seen in Figure 2a that the grain size of received ZK60 alloy is about 29 μm and uneven, while the grains of annealed ZK60 alloy have a certain degree of growth, and the average grain size is about 35 μm. However, the size is still uneven because recrystallization occurs during heat treatment and smaller grains are formed, as shown in Figure 2b.

**Figure 3:**
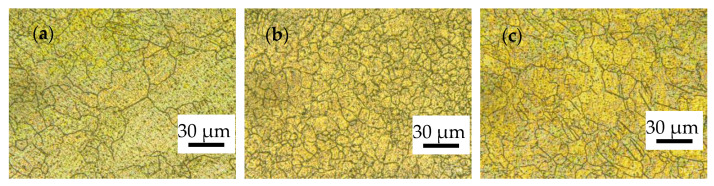
OM of ZK60 alloy rolled with different RSR in path A: (**a**) 1:1.15; (**b**) 1:1.2; (**c**) 1:1.5.

Figure 3 shows the OM of the alloy rolled with a different RSR under path A. It can be seen in the figure that the grain size is refined with the increase in RSR, and the average grain size of 30 μm (as shown in Figure 3a) is refined to about 23 μm (as shown in Figure 3b,c). By comparing Figure 3b,c, it can be found that the microstructure uniformity of the alloy rolled with an RSR of 1:1.2 is better. In Figure 3c (marked in white), it can be seen that a small number of intersecting twins appear in the microstructure of the alloy rolled with an RSR of 1:1.5.

**Figure 4:**
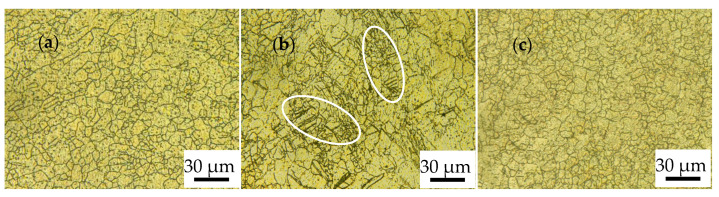
OM of ZK60 alloy rolled with different RSRs in path B: (**a**) 1:1.15; (**b**) 1:1.2; (**c**) 1:1.5.

Figure 4 shows the microstructure of the alloys rolled with different RSRs under path B. It can be seen that the grain size of the alloy decreases first and then increases with the increase of the RSR. An average grain size of about 14 μm of the alloy rolled with an RSR of 1:1.2 was obtained and intersecting twins appear on larger grains, as shown in Figure 4b. Meng et al. [22] used asynchronous rolling to roll AZ31 alloys and found that a too-high RSR would cause slippage between the roll and the sheet, resulting in reduced shear deformation of the alloy due to sliding friction. Due to the existence of the slippage phenomenon and the excessive rotation angle in the rolling direction of each pass under path B, the shear stress acting on the alloy is not uniform enough, resulting in unrefined grains and uneven distribution of intersecting twins (marked in white).

**Figure 5:**
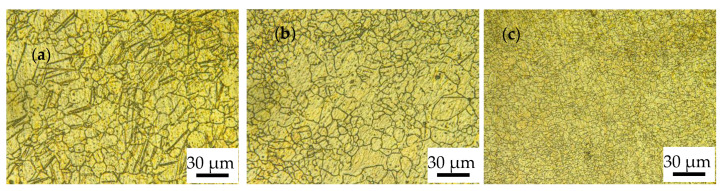
OM of ZK60 alloy rolled with different RSRs in path C: (**a**) 1:1.15; (**b**) 1:1.2; (**c**) 1:1.5.

Figure 5 shows the microstructure of the alloy rolled with different RSRs under path C. It can be seen in the figure that the grain size was significantly refined with the increase of RSR, the average grain size of the alloy decreased from about 27 μm (Figure 5a) to about 13 μm (Figure 5c), and the grains were significantly refined. This is because cross-rolling makes the alloy start non-basal slip, such as prismatic slip and pyramidal slip, during deformation [23,24]. With the increase in the RSR and the change of the rolling direction, the shear stress acting on the sliding surface of each grain increases and the direction changes constantly, so it is necessary to coordinate the deformation through twinning and dynamic recrystallization during the MAR process. Dynamic recrystallization behavior is an important mechanism of grain refinement during magnesium alloy deformation [25]. Due to the activation of the non-basal slip system, the proportion of dynamic recrystallization increases and the grain refinement is promoted. Therefore, the C-1.5 process obtained an average grain size of about 13 μm by cross-rolling with a large RSR.

### 3.2. Mechanical Properties of MARed ZK60 Magnesium Alloys

**Figure 6:**
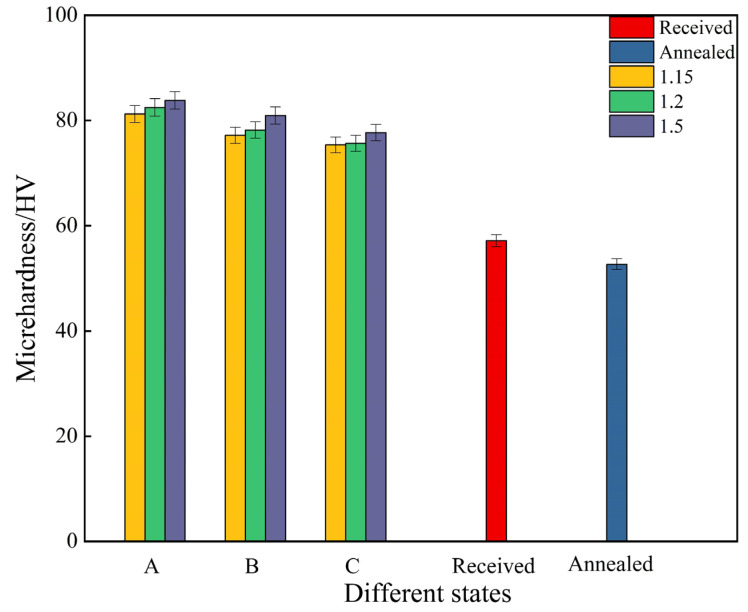
Hardness values of ZK60 alloys under different states.

Figure 6 shows the hardness values of ZK60 alloys under different states. It can be seen that the hardness of the MARed alloys has been significantly improved, much higher than that of the received and annealed ZK60 alloys. However, it can be seen that the changes in RSR and rolling path have no significant impact on the hardness of the alloys.

**Figure 7:**
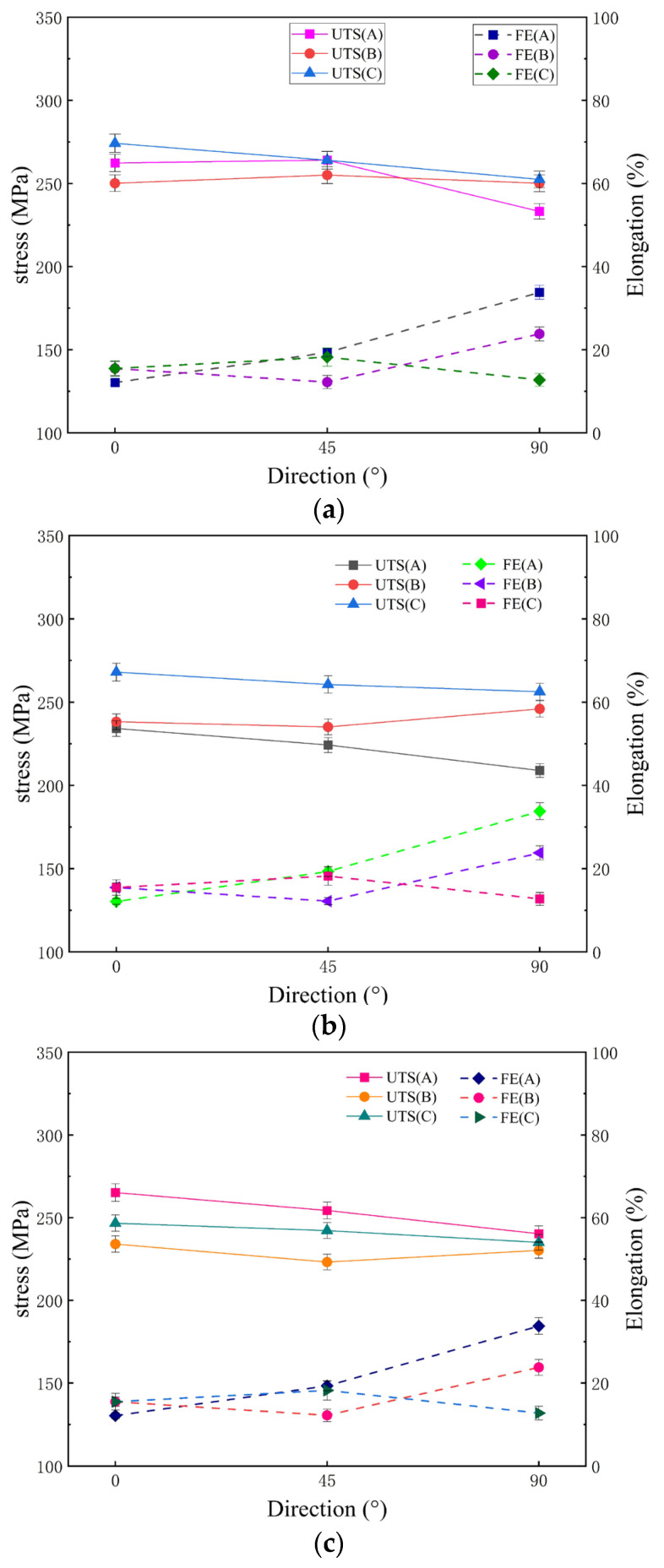
UTS and FE of MARed ZK60 alloys (UTS: Ultimate tensile strength; FE: Elongation after fracture): (**a**) 1:1.15; (**b**) 1:1.2; (**c**) 1:1.5.

Figure 7 shows the room temperature tensile properties of the MARed ZK60 alloy in the rolling direction (0° direction), 45° direction, and transverse direction (90° direction). From Figure 7a (RSR of 1:1.15), it can be seen that the change of rolling path has little effect on the elongation after fracture (FE). The ultimate tensile strength (UTS) of the ZK60 alloy sheet rolled under path C is much greater than that under paths A and B, and the UTS under path C in different tensile directions has no obvious change, showing better isotropy.

It can be seen in Figure 7b that under the rolling condition of RSR of 1:1.2, the UTS of the alloy under path B and path C is close, and both are slightly higher than that obtained under path A. Observing the FE in Figure 7b, it is found that rolled under path B and path C, the alloy shows good isotropy, and the FE does not change significantly in different tensile directions, but the FE of the alloy rolled under path A is 20% different in 0° and 90° tensile directions, and the anisotropy is significant. The tensile strength of the alloys rolled under path B increases in the 90° tensile direction, as shown in Figure 7c (RSR of 1:1.5). The elongation of the alloys rolled under paths B and C in the 0° tensile direction is slightly higher than that under path A, and the three rolling paths show great differences in the 90° tensile direction. A higher elongation was obtained in the alloys rolled under path A, which is about 15% higher than the lowest elongation obtained in the alloys rolled under path C.

Based on the above discussion on the microstructures and mechanical properties of ZK60 alloys, it can be seen that MAR processing can refine the grains and improves microstructure uniformity. The change of rolling path and RSR had a significant impact on the microstructure and mechanical properties of the alloy. Very fine and uniform equiaxed grains were obtained in the alloy prepared by the C-1.5 process, and the tensile strength and elongation of the alloy reached maximum values.

## 4. Discussion

**Figure 8:**
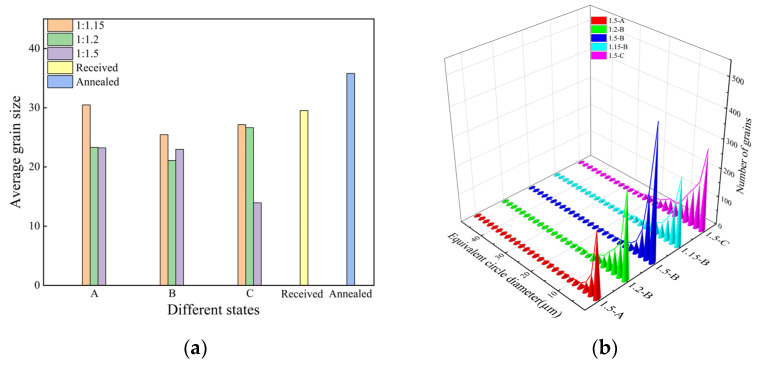
(**a**) Average grain size and (**b**) grain size distribution (according to EBSD obtained by data analysis) of MARed ZK60 alloys.

In order to explore how the C-1.5 process can improve the properties of ZK60 alloys, factors such as shear modes of different rolling processes, proportion of high-angle grain boundaries, proportion of randomly oriented grains, and texture intensity are compared and analyzed. The three rolling processes shown in Table 1 can be divided into two categories according to the shear mode. One is that the alloys rolled under path A, the upper and lower surfaces of the sample, were kept in the same position as the slow and fast rollers, and the shear deformation occurred on the same plane; another method is that the alloys rolled under paths B and C, where the side of the rolled sheet that came into contact with the slow roller in the previous pass needed to be flipped 180° to make contact with the fast roller before proceeding to the next pass. The shear deformation experienced by the alloy will occur in different planes and directions, and the alloy can undergo more uniform shear strain and is more conducive to enhancing grain refinement ability. Figure 8 shows the average grain size and grain size distribution of MARed ZK60 alloys. The grain refinement effect of the alloy rolled by the C-1.5 process is obvious, as shown in Figure 8a, and the grain uniformization effect also is the best, as shown in Figure 8b. The proportion of grains less than 20 μm is much higher than that of the alloys rolled by other processes, and the distribution of grain quantity in each size is more uniform, showing good uniformity. Based on the above results, the alloys rolled by five rolling processes, C-1.5, A-1.5, B-1.5, B-1.2, and B-1.15, were selected for EBSD testing.

Due to the hcp of the ZK60 alloy with few internal slip systems, twinning has become an important way for the alloy to coordinate deformation during the rolling process. Intersecting twins can be found in the microstructure of MARed ZK60 alloys in Figure 3, Figure 4 and Figure 5. The alloys rolled by B and C processes are subjected to shear stress in multiple directions, resulting in the intersecting twins having no specific direction, and the secondary twins are prone to occur inside these twins due to coordinated deformation, which provides a large number of sites for dynamic recrystallization nucleation. In addition, the “segmentation” effect of twin intersection on grains also has the effect of refining grains [26,27]. Dynamic recrystallization, as an important mechanism for grain refinement, can effectively activate the non-basal slip system, thereby improving the plasticity of the alloy, further promoting the occurrence of dynamic recrystallization. The original twins were covered and uniformly distributed, and fine equiaxed grains were formed finally. The shear stress acting on the alloy rises when the RSR increases, causing subgrains to rotate, and the orientation difference angle of low-angle grain boundaries (LAGBs) gradually increases, ultimately forming high-angle grain boundaries (HAGBs). During the hot working process of alloys, the nucleation of new grains comes from the transformation of LAGBs for subgrains to HAGBs [28]. The comparison of the HAGBs ration in Table 3 shows that the microstructure of the alloy rolled by the C-1.5 process has evolved into fine grains surrounded by HAGBs.

Random oriented grains generated by dynamic recrystallization of the MARed alloys can result in the weakening of basal texture intensity. Table 3 also shows the ratio of random orientation grains in MARed ZK60 alloys. It can be seen that the ratio of randomly oriented grains in the alloys rolled by the C-1.5 and B-1.5 processes is higher than that rolled by the A-1.5 process due to the alternating changes in rolling direction. The microstructure of the alloy rolled by the C-1.5 process exhibits alternating distribution of deformed grains, as shown in the IPF diagram in Figure 10 (marked by white circles), and can weaken the texture intensity of the alloy.

**Figure 9:**
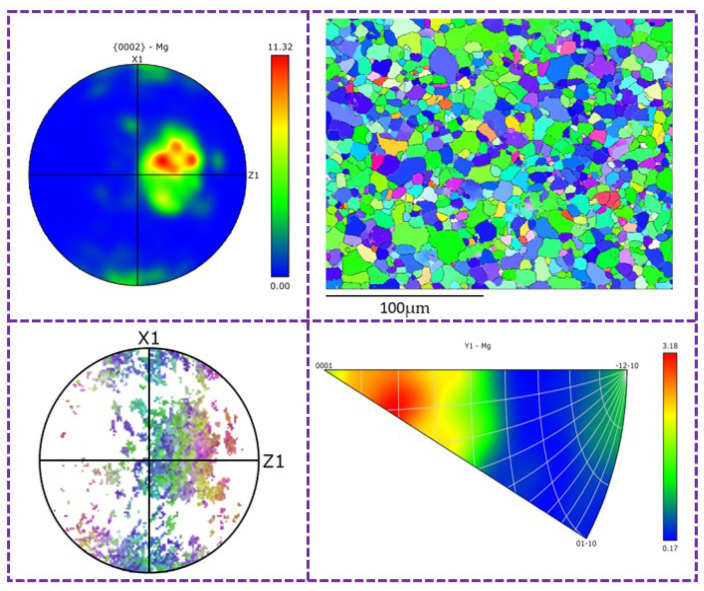
Segment IPF diagram of correlation polar diagram of ZK60 alloy rolled under C-1.5 process.

**Figure 10:**
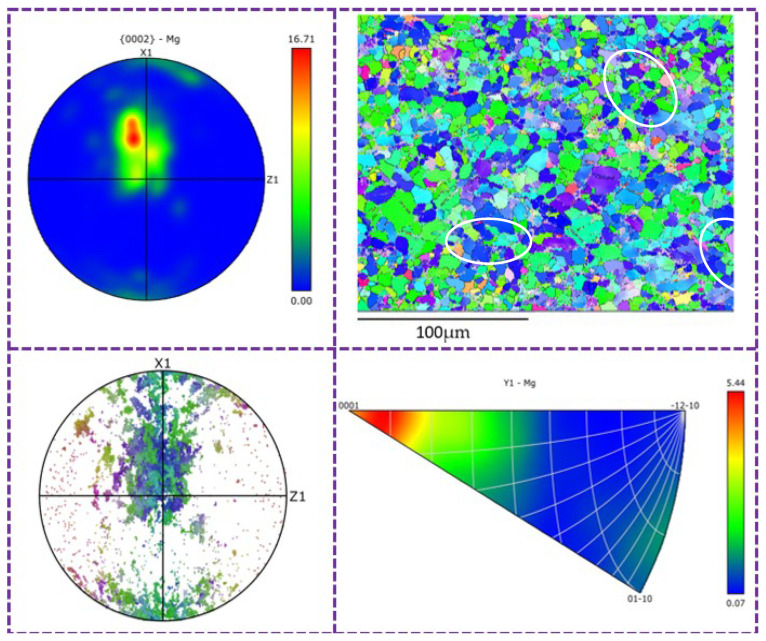
Segment IPF diagram of correlation polar diagram of ZK60 alloy rolled under B-1.15 process.

Figure 9 and Figure 10 show the segmented IPF diagram of the ZK60 alloy-related polarity diagram rolled by the b-1.15 and c-1.5 processes, respectively. Twinning can also be seen in Figure 9. With the increase in the frequency of path c direction change and the increase in the allometric ratio, it can be found that as dynamic recrystallization has basically completed in the alloy (as shown in Figure 10), twins basically disappear. In the figure, the grains with red color are those whose c-axis is parallel to the (0001) base plane, while the grains with green and blue color represent those that deviate from the (0001) base plane. In Figure 10, we can see that the number of red grains in the microstructure is significantly reduced because of the occurrence of complete dynamic recrystallization and a large number of “swallowing” twins, thus obtaining a more uniform microstructure. The average grain size is reduced, and the base plane texture strength of the alloy is also reduced to 13.72. The decrease of average grain size leads to the number of grains and grain boundaries being increased within the microstructure. According to the principle of fine grain strengthening, the strength of the alloy will increase accordingly, which is consistent with the results obtained in mechanical tensile tests. The mechanical properties of the alloy are optimal when rolled by C-1.5 [29,30].

The random grain orientation ratio of ZK60 alloys rolled by the C-1.5 process is increased, weakening the texture intensity of the alloy. According to Schmid’s law, in magnesium-based materials, weaker basal textures can lead to softening effects, and the basal-slip system is more easily activated in grains with higher Schmid factors, which leads to more plastic materials [31]. Table 4 shows the Schmid factor ratios for the MARed ZK60 alloys (where the Schmid factor ratio ranges from 0.4 to 0.5). It can be observed that the alloys rolled by the C-1.5 process obtained the highest Schmid factor values. Therefore, ZK60 alloys rolled by the C-1.5 process can achieve good plasticity.

## 5. Conclusions

ZK60 magnesium alloy sheets rolled by MAR were combined with different RSR and rolling paths, and the effects of different processes on the microstructure evolution and mechanical properties of alloy were studied. The conclusions can be drawn as follows:

(1) The MAR process can effectively refine and uniform the grain size of the ZK60 alloy, reduce the basal texture intensity, improve mechanical properties, and reduce anisotropy, which will effectively improve formability of the ZK60 alloy. After rolling at 400 °C through the C-1.5 process, the strength and plasticity of ZK60 magnesium alloy reached the maximum values, and the anisotropy of mechanical properties was significantly improved.
(2) Grains were refined with the RSR increases during hot rolling due to the shear stress acting on the alloys’ increases and led to the rotation of subgrains and the transition from LAGBs to HAGBs, which promotes the occurrence of dynamic recrystallization and small grain formation. Intersecting twins with cross-orientation obtained in the MARed alloys subjected to shear stress from different directions are prone to generating secondary twinning internally during coordinated deformation, which also promotes the occurrence of dynamic recrystallization. The “segmentation” effect of twins intersection also refines grains. Optimized MAR processing (C-1.5) can improve the non-uniformity of alloy microstructure and make the grain size more uniform.
(3) The randomly oriented grains generated by dynamic recrystallization of the MARed alloys can weaken the strong basal texture generated during the rolling process, and improve the anisotropy of alloy mechanical properties.

## Figures and Tables

**Table 1:** Three different rolling paths used in this work. RSR: 1:1.15 = 12 rpm/10.43 rpm; 1:1.2 = 12 rpm/10 rpm; 1:1.5 = 12 rpm/8 rpm.

Rolling Path	1st Pass	Path Changes	2nd Pass
Path A	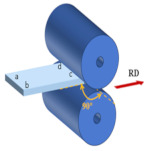	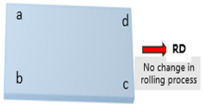	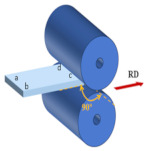
Path B	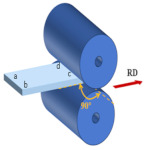	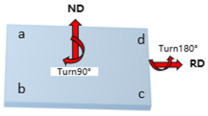	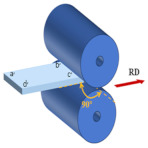
Path C	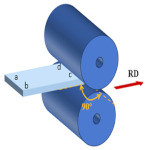	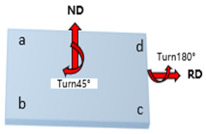	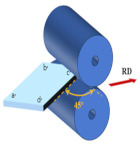

**Table 2:** Chemical composition of experimental ZK60 magnesium alloy (mass fraction, %).

Element	Zn	Zr	Mn	Ni	Al	Cu	Fe	Si	Mg
Content	5.7	0.3~0.6	0.10	0.01	0.05	0.05	0.05	0.05	Bal.

**Table 3:** The random orientation ratio and high-angle grain boundary ratio of the MARed alloy grains.

Rolling Process	Random Orientation Ratio (%)	High Angle Grain Boundary (%) (>15°)
A-1.5	46.30%	49.30%
B-1.2	47.00%	70.50%
B-1.5	50.60%	70.40%
B-1.15	75%	77.10%
C-1.5	82.50%	89.20%

**Table 4:** Schmid factor ratio for MARed ZK60 alloys (Schmid factor ratio ranges from 0.4–0.5 here, SF come from <0001> basal plane).

Rolling Process	Schmid Factor
A-1.5	0.306
B-1.15	0.368
B-1.2	0.211
B-1.5	0.222
C-1.5	0.443

## Data Availability

Data are contained within the article.

## References

[1] Sun Y., Chi C.C., Li L.C. (2017). Effect of anisotropic of ZK60 magnesium alloy sheet on its earing behavior during deep drawing process. J. Plast. Eng..

[2] Tan J., Ramakrishna S. (2021). Applications of Magnesium and Its Alloys: A Review. Appl. Sci..

[3] Kaya A.A. (2020). A Review on Developments in Magnesium Alloys. Front. Mater..

[4] Kim K.H., Okayasu K., Fukutomi H. (2015). Influence of the Initial Texture on Texture Formation of High Temperature Deformation in AZ80 Magnesium Alloy. Mater. Trans..

[5] Huang B., Yan H.G., Chen J.H. (2018). Effects of Rolling Process on Microstructure and Tensile Properties of ZK60 Magnesium Alloy. Mater. Mech. Eng..

[6] Azghandi S.H.M., Weiss M., Arhatari B.D., Barnett M.R. (2020). Grain size and void formation in Mg alloy AZ31. J. Alloys Compd..

[7] Xin S.H., Suzuki K., Yong J.K. (2007). Effects of differential speed rolling on microstructure and mechanical properties of AZ31 magnesium alloy. Materials Science Forum.

[8] Wang W., Chen W., Zhang W. (2018). Effect of deformation temperature on texture and mechanical properties of ZK60 magnesium alloy sheet rolled by multi-pass lowered-temperature rolling. Mater. Sci. Eng..

[9] Li L.B., Jiang P., Ren G.M. (2020). Effect of Rolling Route on Microstructure and Properties of ZK60 Magnesium Alloy Sheets. Heat Treat. Met..

[10] Mostaed E., Hashempour M., Fabrizi A., Dellasega D., Bestetti M., Bonollo F., Vedani M. (2014). Microstructure, texture evolution, mechanical properties and corrosion behavior of ECAP processed ZK60 magnesium alloy for biodegradable applications. J. Mech. Behav. Biomed. Mater..

[11] Karparvarfard S.M.H., Shaha S.K., Behravesh S.B., Jahed H., Williams B.W. (2017). Microstructure, texture and mechanical behavior characterization of hot forged cast ZK60 magnesium alloy. J. Mater. Sci. Technol..

[12] Wagner L., Hilpert M., Wendt J. (2003). On Methods for Improving the Fatigue Performance of the Wrought Magnesium Alloys AZ31 and AZ80. Mater. Sci. Forum.

[13] Pustovoytov D., Pesin A., Tandon P. (2021). Asymmetric (Hot, Warm, Cold, Cryo) Rolling of Light Alloys: A Review. Metals.

[14] Peláez D., Isaza C., Meza J.M., Fernández-Morales P., Misiolek W.Z., Mendoza E. (2015). Mechanical and microstructural evolution of Mg AZ31 alloy using ECASD process. J. Mater. Res. Technol..

[15] Kim B., Baek S.M., Lee J.G., Park S.S. (2017). Enhanced strength and plasticity of Mg–6Zn–0.5Zr alloy by low-temperature indirect extrusion. J. Alloys Compd..

[16] Chen B., Lin D.L., Zeng X.Q. (2010). Effect of Asymmetric Cross Rolling on Microstructure and Mechanical Properties of AZ31 Magnesium Alloy. Mater. Mech. Eng..

[17] Lin N., Liu S.F., Liu Y.H. (2018). Microstructure and texture evolution of pure tantalum during asymmetrical rolling and subsequent annealing treatment. J. Chin. Electron. Microsc. Soc..

[18] Chu Z.J., Bo L., Wang W.H., Du Y., Sun Y. (2021). Hot deformation behavior and recrystallization of 6061 aluminum alloy. Rare Met. Mater. Eng..

[19] Jia W.P., Hu X.D., Zhao H.Y., Ju D.Y., Chen D.L. (2015). Texture evolution of AZ31 magnesium alloy sheets during warm rolling. J. Alloys Compd..

[20] Hadadzadeh A., Wells M.A., Shaha S.K., Jahed H., Williams B.W. (2017). Role of compression direction on recrystallization behavior and texture evolution during hot deformation of extruded ZK60 magnesium alloy. J. Alloys Compd..

[21] Al-Samman T., Gottstein G. (2008). Dynamic Recrystallization During High Temperature Deformation of Magnesium. Mater. Sci. Eng..

[22] Meng Q., Cai Q.W., Jiang H.T. (2011). Effect of Differential Speed Rolling on Static Recrystallization and Grain Refinement of AZ31 Magnesium Alloy. Chin. J. Eng..

[23] Jin S.C., Kim Y.J., Lee D.H., Han S.H., Jo S., Park S.H. (2024). Recrystallization behavior and microstructure evolution of Mg–5Bi–3Al alloy during very high-speed extrusion. J. Mater. Sci. Technol..

[24] Robson J.D., Henry D.T., Davis B. (2009). Particle effects on recrystallization in magnesium–manganese alloys: Particle-stimulated nucleation. Acta Mater..

[25] Lee S.W., Park S.H. (2020). Static recrystallization mechanism in cold-rolled magnesium alloy with off-basal texture based on quasi in situ EBSD observations. J. Alloys Compd..

[26] Mo C., Kontsos A. (2018). Twinning contributions to strain localizations in magnesium alloys. Mater. Sci. Eng. A.

[27] Mostaed E., Fabrizi A., Dellasega D., Bonollo F., Vedani M. (2015). Grain size and texture dependence on mechanical properties, asymmetric behavior and low temperature superplasticity of ZK60 Mg alloy. Mater. Charact..

[28] Gui Y., Ouyang L., Cui Y., Bian H., Li Q., Chiba A. (2021). Grain Refinement and Weak-textured Structures Based on the Dynamic Recrystallization of Mg–9.80Gd–3.78Y–1.12Sm–0.48Zr Alloy. J. Magnes. Alloys.

[29] Hoseini-Athar M.M., Mahmudi R., Babu R.P., Hedström P. (2021). Microstructure and superplasticity of Mg–2Gd–xZn alloys processed by equal channel angular pressing. Mater. Sci. Eng. A.

[30] Fakhar N., Sabbaghian M., Nagy P., Fekete K., Gubicza J. (2021). Superior low-temperature superplasticity in fine-grained ZK60 Mg alloy sheet produced by a combination of repeated upsetting process and sheet extrusion. Mater. Sci. Eng. A.

[31] Ko Y.G., Hamad K. (2018). Structural Features and Mechanical Properties of AZ31 Mg Alloy Warm-deformed by Differential Speed Rolling. J. Alloys Compd..

